# Music Therapy Intervention in an Open Bay Neonatal Intensive Care Unit Room Is Associated with Less Noise and Higher Signal to Noise Ratios: A Case-Control Study

**DOI:** 10.3390/children9081187

**Published:** 2022-08-08

**Authors:** Shmuel Arnon, Shulamit Epstein, Claire Ghetti, Sofia Bauer-Rusek, Riki Taitelbaum-Swead, Dana Yakobson

**Affiliations:** 1 Department of Neonatology, Meir Medical Center, Kfar Saba 4428163, Israel; 2 Sackler Faculty of Medicine, Tel Aviv University, Tel Aviv 6329302, Israel; 3 School for Creative Arts Therapies, University of Haifa, Haifa 3498838, Israel; 4 GAMUT–The Grieg Academy Music Therapy Research Centre, University of Bergen, 5020 Bergen, Norway; 5 Department of Communication Disorders, Ariel University, Ariel 40700, Israel; 6 Music Therapy Department, Aalborg University, 9220 Aalborg, Denmark

**Keywords:** infants, NICU, noise, music therapy, preterm

## Abstract

Background: Noise reduction in the Neonatal Intensive Care Unit (NICU) is important for neurodevelopment, but the impact of music therapy on noise is not yet known. Objective: To investigate the effect of music therapy (MT) on noise levels, and whether individual MT (IMT) or environmental MT (EMT) increases meaningful signal-to-noise ratios (SNR). Study design: This case-control study was conducted in a level III NICU. Noise levels were recorded simultaneously from two open bay rooms, with a maximum of 10 infants in each room: one with MT and the other without. MT sessions were carried out for approximately 45 min with either IMT or EMT, implemented according to the Rhythm Breath and Lullaby principles. Noise production data were recorded for 4 h on 26 occasions of EMT and IMT, and analyzed using R version 4.0.2 software. Results: Overall average equivalent continuous noise levels (Leq) were lower in the room with MT as compared to the room without MT (53.1 (3.6) vs. 61.4 (4.7) dBA, *p* = 0.02, *d* = 2.1 (CI, 0.82, 3.42). IMT was associated with lower overall Leq levels as compared to EMT (51.2 vs. 56.5 dBA, *p* = 0.04, *d* = 1.6 (CI, 0.53, 1.97). The lowest sound levels with MT occurred approximately 60 min after the MT started (46 ± 3.9 dBA), with a gradual increase during the remaining recording time, but still significantly lower compared to the room without MT. The SNR was higher (18.1 vs. 10.3 dBA, *p* = 0.01, *d* = 2.8 (CI, 1.3, 3.86)) in the room with MT than in the room without MT. Conclusion: Integrating MT modalities such as IMT and EMT in an open bay NICU room helps reduce noise. Both MT modalities resulted in higher SNR compared to the control room, which may indicate that they are meaningful for the neurodevelopment of preterm infants.

## 1. Introduction

Noise is an unwanted, unpleasant, or unexpected sound. In a Neonatal Intensive Care Unit (NICU) setting, it is generated from multiple sources such as equipment, alarms, telephones, crying infants, and family and staff conversations [1,2,3,4]. Infants in a NICU are subjected to stressful, unprotected, high-intensity noise [5]. Noise levels in NICUs may reach up to 120 dB [6], and they frequently exceed the maximum 45 dB level endorsed by the American Academy of Pediatrics (AAP) [7]. Noise levels are much higher in open bay rooms as compared to private rooms [8], making this hazard even more pronounced in NICU settings. Exposing preterm infants to sustained, intense noise can be damaging and may be related to stress responses, physiological and autonomic changes, a lack of sleep, altered endocrine and metabolic responses, and hearing deficits [9,10,11]. Noise levels can be reduced in various ways, including the architectural design of the NICU [12], decreasing sound from equipment and alarms [13], educating personnel and families to reduce the sound levels they create [14], applying sound-reducing devices to preterm infants, or a combination of these methods [15]. In terms of architectural structures designed to reduce noise levels, private rooms and/or sound absorbent material on walls and ceilings near clinical activity areas are part of the recommended measures in the design of a modern NICU [16]. Even though modern NICU equipment is designed to generate low noise levels during operation and alarms, high noise levels were recorded from a variety of equipment alarms [4]. A systematic review [17] investigating whether sound reduction results in better outcomes for preterm infants found an advantage in one small study that used earplugs to reduce the sound levels that reached preterm infants. There was a trend for better growth at 34 weeks postmenstrual age. Using both eye goggles and earmuffs to reduce preterm infants’ exposures to light and noise was not recommended for neonatal practice [15]. Incubators may potentially offer some attenuation against direct external noise, but a few studies have shown that this is very limited for preterm infants, who need repeated opening and closing of the incubator ports, which results in high noise levels [10]. Therefore, the strategies most often used to reduce perceptual sound levels are insulation and isolation [18]. These approaches fall short in that they only address the reduction in stressors, and they do not contribute toward creating developmentally appropriate auditory stimuli.

Music-based interventions in the NICU are attuned to environmental sound, to make it less intimidating and more pleasant for the baby, the parents, and the personnel dealing with the high levels of unpleasant sound [19,20,21]. A recent systematic review of music-based interventions in the NICU suggested that they were associated with significant improvements in pain relief and some physiological and behavioral parameters, such as cardiac and respiratory function, weight gain, feeding behaviors, and quiet-alert and sleep states [20].

A few explanations were suggested regarding the beneficial effects of music-based interventions in the NICU. Music therapy (MT) had a strong influence on parents’ relaxation, improved bonding, fostered development, and promoted better outcomes among preterm infants, including improved vital signs, and better feeding and sleep during MT intervention [22,23,24]. Improved breastfeeding rates and volumes due to positive conditioning via MT were also among the possible explanations for the mechanisms involved [23,24]. Because many cognitive elements of music are processed simultaneously or sequentially by both cerebral hemispheres, repeated listening and music training stimulate neurologic development in preterm infants [25].

The effect of music-based interventions on noise reduction in the NICU has not been evaluated in depth. Music can mask ambient environmental sound, and can alleviate the negative consequences of noise, such as fatigue, stress, hyper-alertness, or startle responses.

One of the active forms of music interventions is MT. It can be implemented in the NICU as environmental music therapy (EMT) and individual music therapy (IMT). EMT has been defined as “a human-centered, trauma-informed intervention that uses the associative properties of live music to modulate patients’, caregivers’ and staff’s perceptions of the hospital milieu as a potentially hostile environment” [26]. In EMT, the music therapist plays or sings live improvised music in the open NICU space, in attunement to the dynamics, movements, and sounds of the physical environment, with the goal of entraining their rhythm, pitch and affect to the surrounding sounds. The playing may develop into a defined song. The first pilot study on EMT showed that it had positive influences in stabilizing infants’ heart and respiratory rates, and in raising the staff’s awareness regarding their personal contributions (such as talking) to elevated noise levels in the NICU [27].

Individual music therapy (IMT) is usually conducted with a music therapist at bedside to support the co-regulation and bonding of the parent and preterm infant, and to reduce their stress. During IMT, music therapists support caregivers’ vocalizing or singing to their preterm infants in a developmentally appropriate manner, while observing the infant’s behavior state and responses, to facilitate early contact and bonding. When caregivers are unavailable for various reasons, IMT may be conducted with a music therapist and infant only. IMT studies show that MT benefits preterm infants’ physiological outcomes such as respiratory rate [28], sleep, and feeding volumes, as well as short-term neurological outcomes [25], and decreases infants’ stress levels [29] and maternal anxiety [30]. A well-known technique for selecting songs is the “Song of Kin” [31], which emphasizes the importance of the family’s musical heritage. In it, the therapist helps adapt parents’ preferred songs into a lullaby style. Another fundamental MT principle is infant-directed singing [32], which focus on live singing to infants based on attunement and entrainment to signals of communication and responses in the moment.

As noise is an unwelcome sound that has undesirable effects on the physiologic state of preterm infants; MT may improve stability. The objectives of the current study were twofold. First, we evaluated the noise levels and the signal-to-noise ratios (SNR) in an open bay NICU room during and after MT sessions, and compared them simultaneously to the level of noise and SNR in a similar room without MT, and second, to evaluate the level of noise during IMT vs. EMT.

## 2. Methods

### 2.1. Setting

This case-control study was conducted in the NICU at Meir Medical Center, Kfar-Saba, Israel; a level III certified Neonatal Individualized Developmental Care and Assessment Program certification unit. It took place from March to July 2017, and was approved by the Meir Medical Center Institutional Review Board (0228-17-MMC) and conducted according to the principles of the Declaration of Helsinki.

The parents of each infant hospitalized in the NICU were informed that noise levels are regularly monitored for information and care. Sound is defined as vibrations that travel through air or another medium that can be heard when they reach a person’s or animal’s ears. Noise is defined as unwanted sound [33]. Parents provided written informed consent allowing us to use the acoustic data anonymously.

**Figure 1:**
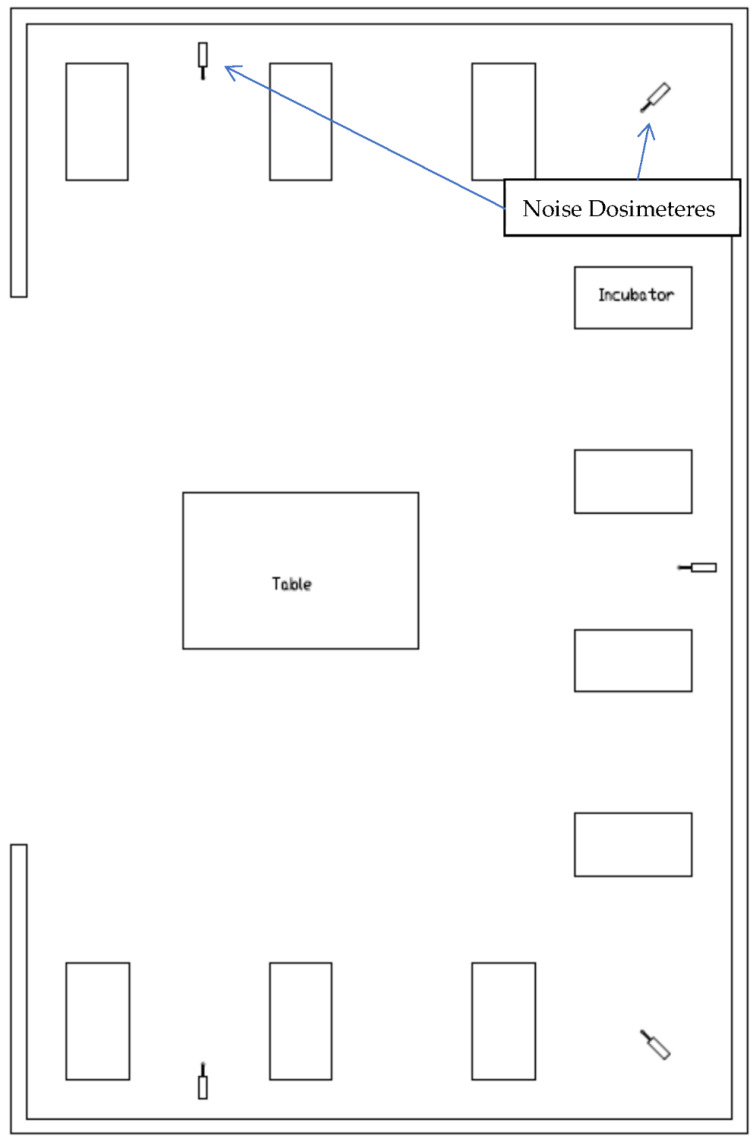
Layout of open bay NICU with 10 incubators.

The NICU treats more than 400 neonates annually. It is composed of 2 neonatal care rooms with an open bay design. The rooms are 13 × 10 m. The average daily census is 8–10 infants per room. The nurses have a workstation in the center of the room and the incubators are situated along the walls (Figure 1). Both rooms (1 with MT (case) and 1 without MT (control) had noise dosimeters in place throughout the study.

Each patient care area is approximately 1.5 m apart. One nurse takes care of 3 neonates, usually one requiring ventilation and two without. Two parents per neonate are allowed to help the nursing staff, assist with skin-to-skin contact (SSC) or other tasks, simultaneously, with no limitations on visiting hours. Mobile phones or other sound source devices are not allowed in the room. Each incubator is separated by a curtain. The walls consist of painted drywall, and one wall contains glass windows that occupy less than half of the wall. The floor is covered with vinyl tiles and the ceiling with acoustic tiles. The walls and floor do not have any noise absorption or damping properties.

### 2.2. Ambient Noise Reduction Measures

Monthly educational sessions regarding the reduction of sources of noise, and methods to reduce ambient noise are conducted. Daily routines reinforcing these behaviors include holding rounds away from the incubator, and nurses conducting handoff 2 m from the patient care area when feasible. Ambient lights are dimmed except when needed for direct patient care. Notices are placed reminding people to limit extraneous noise, as additional cues to support the quiet environment. We do not use noise-sensitive light alarms, as our local experience resulted in habituation to that device. A nurse is assigned as a noise monitor each shift, to remind everyone of their responsibilities regarding reducing noise in each room. Duties include observing the ambient noise level and reminding other providers and parents when these levels increase.

### 2.3. Music Therapy Interventions

MT sessions were carried out for approximately 45 min, during morning or afternoon shifts (9 AM–6 PM), but not during nursing reports. Ambient noise was recorded for 4 h, starting 30 min before a MT session began.

Both EMT and IMT were based on the “First Sounds: Rhythm, Breath and Lullaby” (RBL model) [22,31,34], and facilitated by a music therapist certified in RBL. Prior to the IMT session, an introductory conversation between the therapist and parents was held to assess families’ resources and needs, musical heritage, and preferences. During MT sessions, the mother–infant dyad was in SSC position, as described previously [35]. Parents were guided to reorganize their breathing rates and to gradually synchronize their breathing patterns to their infants’ respiratory rates. The music therapist sitting nearby accompanied them using the Remo Ocean disk, whose sound resembles the intrauterine sound environment. This has previously demonstrated efficacy in promoting breathing and relaxation [22]. Parents were then guided to hum simple melodies repeatedly, that gradually evolved into singing 2 or 3 songs of their choice, adapted to the rhythm of a lullaby. This is known as the “Song of Kin” technique [31]. Humming and singing were accompanied by guitar music if the parents desired. Parents who did not want to sing, listened to the therapist performing a song of their choice.

The introduction of a person-directed dynamic music “soundtrack” through EMT enhances feelings of control and normality among parents and infants [36]. The concept of creating an environment through the use of sound and music engenders feelings of safety and relaxation [37]. The music therapist first assessed the current sound environment, including human and equipment noise, as well as the current dynamics evident in the room (number of families and personnel, interactions and their type, medical procedures being performed, and the emotional atmosphere related to the health status of the patients and their families, or the medical staff. The music therapist then began to play simple tunes on the guitar, aiming to engage the music with the sounds currently present (for example, synchronizing or reflecting the rhythms and beeps produced from monitors and gradually developing this improvisation with certain musical motifs such as known lullabies, and slowly adding vocals and singing lyrics. The closure of the EMT session included another dynamic, gradually cueing the end of musical peace with elements such as a reduction in vocals, notes, and rhythms. Both types of MT were presented for 30–45 min, 3 times a week, with one therapist available at a time. They were applied using a randomly generated sequence of IMT or EMT.

### 2.4. Outcomes and Noise Level Measurements

The primary outcome was the ambient noise level in the open bay room of the NICU during MT sessions (study arm) compared to the room without MT (control arm). Data were gathered regarding gestational age at birth, age at recruitment, number of infants per room per session, how many were ventilated (a measure of medical severity) and ambient sound.

Noise levels in both open bay rooms were recorded simultaneously using Spark 703+, 705+ and 706RC Type II Noise Dosimeters (Larson Davis Laboratories, Provo, UT, USA) to record noise coming from all sources around the dosimeter area. We used noise dosimeters as special-purpose sound level meters, due to their small dimensions, their ability to record and accumulate noise data for 4–8 h, their safety features, and their ease in analyzing the output data. The dosimeters were situated on a countertop, and microphones were taped to posts located between two isolates. Figure 1 shows the placement of the dosimeters. They were on the A-weighted, fast detector setting (0.125 s interval), with a 1 s sample interval, 30 dB gain, and 3 dB exchange rate, according to the manufacturer’s instructions. Dosimeters were calibrated before the recording sessions. Pre- and post-calibration information were according to the American National Standards Institute [38]. The average equivalent continuous sound level (Leq) and the A-weighted sound levels exceeding 10%, 50%, and 90% of the time (L10, L50, and L90, respectively) were calculated for the duration of the recording period in each room. The SNR L50 dBA was calculated (level of acoustic event − L50 = SNR L50). The principal of calculating SNR is involves subtracting an estimate of the average noise spectrum from the noisy signal spectrum. The noise spectrum is estimated and updated from the periods when the signal is absent and only the noise is present. The assumption is that the noise is a stationary or slowly varying process, and that the noise spectrum does not change significantly between the update periods [39]. Data were downloaded by an independent person who did not know which were the study and control arms, and exported via Blaze v 6.1.1 software (Larson Davis Laboratories, Provo, UT, USA). Recordings lasted 4 h, starting one-half hour before the IMT or EMT sessions.

### 2.5. Power Analysis

Our research question was, “Does applying MT in an open bay NICU room result in different noise and SNR levels, compared to a similar NICU room without MT?” The primary endpoint was to reduce the overall Leq of the noise level in the open bay room from 60 dBA, a mean noise level found in multiple NICU studies [40], to 49 dBA, with an alpha error of 0.05 and 80% power, using Cohen’s d classification [41] This calculation indicated that we should perform 26 measurements to determine a difference between rooms.

### 2.6. Statistics

Data are presented as numbers for nominal variables, and as mean ± standard deviation (SD) for normally distributed continuous variables, or as medians and interquartile range (IQR) when the distribution was not normal (according to the Shapiro–Wilk test). Effect size (*d*) with confidence interval (CI) was calculated. Differences between the study groups: the noise levels of open bay NICU rooms with or without MT were analyzed using a t-test for parametric variables and a chi-squared test for categorical variables. Data were analyzed using R Version 4.0.2 software (R Foundation for Statistical Computing; Vienna, Austria).

## 3. Results

### 3.1. Infant Characteristics

No significant differences in gestational age, post-natal age at study entry, or mean number of infants per session were documented between the two rooms (Table 1, all *p* > 0.05). Medical severity, measured by the need for ventilation, which might produce noise, was not different between the study and control rooms (Table 1). Durations of the EMT and IMT sessions were not significantly different (*p* = 0.19).

### 3.2. Noise Levels and Acoustic Events

Acoustic data for the rooms with and without IMT and EMT are shown in Table 2. The Leq for the 4 h duration of the noise measured in the room with MT was significantly lower than the noise level in the room without MT (53.1 (3.6) vs. 61.4 (4.7) dBA, respectively; *p* = 0.02, *d* = 2.1 (0.82, 3.42). Other measurements concerning the variables of noise levels in a space such as L90, L50, and L10 showed similar results, with lower levels in the room with MT as compared to the open room without MT (Table 2).

The average SNR L50 of events with MT was higher in the room with MT as compared to the room without MT (18.1 (6.1) vs. 10.3 (4.2), respectively; *p* = 0.01, *d*= 2.8 (1.3, 3.86)). The noise levels in both rooms exceeded the maximum acceptable level of 45 dB recommended by the AAP [7]. Table 3 summarizes the acoustic data for the EMT and IMT sessions. IMT was associated with lower noise levels than EMT (51.2 dBA vs. 56.5 dBA, respectively; *p* = 0.04, *d* = 1.6 (0.53, 1.97)).

**Figure 2:**
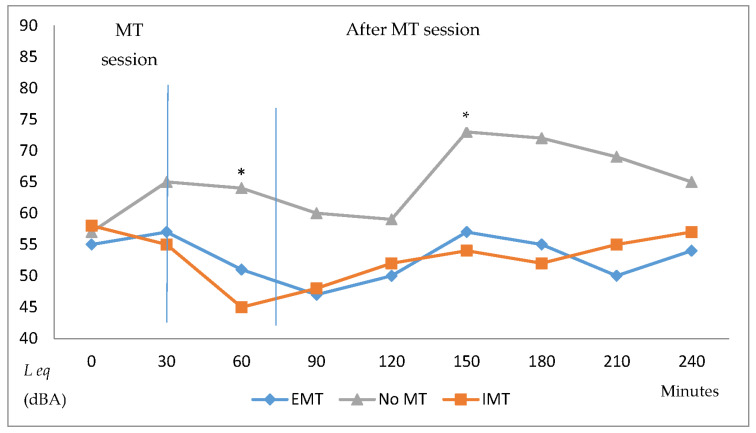
Leq for 4 h, during and after EMT, IMT, and no MT. Leq, approximate mean level of the fluctuating sound over a period; EMT, environmental music therapy; IMT, individual music therapy; MT, music therapy. * *p* < 0.05, compared to IMT and EMT.

Figure 2 shows the noise Leq for 4 h, recorded before, during, and after MT (EMT or IMT) as compared to the room without MT. The noise before MT started showed no differences in Leq. After starting MT, the room with MT had lower noise levels compared to the room without MT, with a significant difference after 30 min. MT lasted for 50 min (Table 1), but after the MT session ended, noise levels were still lower in the room with MT compared to the other room. The lowest sound levels occurred approximately 30 min after the MT started, with gradual increases in sound levels after that, but significantly lower compared to the room without MT.

## 4. Discussion

The current study explored whether MT in an open bay NICU room would result in increased or decreased noise levels, which is important for the well-being and development of preterm infants [4,5,29]. We found that IMT and EMT resulted in lower noise levels during the therapy, and this effect lasted for the entire 4 h of the recording, as compared to a similar room without MT. We also found that IMT resulted in lower noise levels compared to EMT. During MT, the acoustic events, as well as the levels of these events relative to the background noise level, resulted in better SNRs than without MT [39]. Acoustic events with peak noise levels exceeding 84 dBA were greater in the room without MT than in the room with MT. The SNR L50 of the documented acoustic events to the estimated background noise level was higher in the room with MT than in the room without MT. This caused acoustic events to be much more noticeable in the room with MT. Therefore, our study results support the safe implementation of MT as a treatment modality for lowering noise in preterm infants in an open bay NICU room.

Improved architectural designs that consider acoustics, educating medical staff to reduce noise created by conversations, equipment designed to lower noise, and frequent noise or ambient sound measurements can help achieve this goal. We propose using MT to reduce noise proactively. Of note, this study found all sound level measurements to be lower during the MT sessions, but still above the 45 dBA recommended by the AAP as the maximum acceptable level of ambient noise in a NICU.

Considering the recommended developmentally appropriate auditory stimulation for preterm infants, the methodology of our study further extends the current evidence-base, emphasizing the use of parent-led, infant-directed singing [32], and specifically, the key interventions of the RBL model, including Song of Kin [31], the use of the Ocean disk [22], and EMT, as trained and taught by the Louis Armstrong Center for Music and Medicine [26]. Accordingly, the IMT and EMT included live voice and music only, with basic components of attunement and entrainment to the alternating states, needs, and reactions of the infants, parents, and the environment.

The positive impacts of using the maternal voice and listening to music, on the developmental outcomes of preterm infants, parent–infant bonding, and reducing maternal anxiety have been documented extensively [28,29,42,43,44]. Our study may offer additional insights into the possible mechanisms by which maternal singing and live MT support preterm infants’ stability, by adding a critical component of reducing noise and increasing meaningful auditory stimuli.

Furthermore, the NICU environment has been associated with negative long-term outcomes in language development [45]. Although previous standards have focused on the need to create silence in the NICU, this sensory deprivation and elimination of speech and language exposure has been demonstrated to have negative effects on infants’ developing auditory cortex, which can result in language delays [46]. In addition to providing a more tranquil setting for the infants, some sounds, specifically spoken language, have been shown to augment their development [32]. Greater exposure to language during infancy is associated with better language skills and cognitive scores later in life [42]. Since language comprises a small percentage of the sounds to which infants in the NICU are exposed [47], it is important that some language exposure is retained in the NICU. Therefore, interventions that incorporate neurodevelopmentally appropriate sounds, including maternal speech and singing, in the NICU environment could serve both as acoustic maskers and stimuli that benefit infant development. Another advantage of MT is that it can help heighten the awareness of medical personnel and visitors alike, and lead to less noise.

Our data show that MT led to meaningful reductions in noise levels, and indicate that MT was not merely an acoustic distraction. This may be explained by different factors. The positive reactions of staff and family to MT, reported in a previous study [48], may have led to increased attention toward minimizing auditory distraction and reducing unnecessary conversations. Additionally, improvements in infants’ stability, as demonstrated in MT studies with similar IMT intervention [49], may have led to fewer acute events, with a resulting reduction in monitor alarms. Another explanation may be that parents’ increased relaxation due to MT may have led to fewer conversations and prolonged SSC during MT.

We found that IMT resulted in greater noise reduction as compared to EMT. EMT is an environmental intervention that aims to establish a friendly, non-hostile environment; however, this therapy is not individualized. Therefore, in a busy NICU, habituation or low compliance might occur. In our clinical experience, EMT might enhance other positive, yet high arousal, responses by fomenting joy and uplifting moods, which may be followed by a parent or staff member joining the singing or commenting on their reaction to the music. In contrast, IMT is attuned to a specific dyad, and may create a much more intimate atmosphere, which may also influence nearby people to support the family in their unique moment, or increase an awareness of their own contribution to noise levels. Another difference is that in IMT, extra care might be taken by music therapists to keep their voices and instruments to within the AAP recommendation of up to 45 dBA. In EMT, the goal that music and voices will reach the boundary of the open bay may cause voice augmentation. This reaction was described in detail as the “Lombard effect” [50,51].

A major strength of this study is the design, in which noise dosimeters were operated in similar open bay NICU rooms with and without MT, ensuring that the control and study groups were comparable. The NICU populations were similar in terms of illness severity and equipment requirements between the rooms. To the best of our knowledge, this is the first study to measure the noise levels of MT, a rather new treatment modality incorporated in the NICU. In addition, dosimetry was conducted for 4 h each session, which indicated that the effect of MT lasted much longer than the treatment.

The study limitations are that our conclusions are limited to open bay NICUs with up to 10 infants in each room. This may not apply to private or small common rooms. We also could not differentiate the specific sources of noise in each open bay room (with or without MT) because we could not analyze noises from conversations, ringing phones, crying babies, alarms, or equipment sounds, etc., separately. Therefore, no specific conclusions can be drawn as to which specific noise or noises MT reduced. We also did not monitor stress signs in these preterm infants and parents, such as heart rates or stress scores, which would enable us to understand better the mechanisms by which MT reduces noise. As the Leq was lower with MT, we are certain that the effect of MT in reducing noise is not due to an acoustic masking or auditory distraction technique, but a result of the genuine effect of creating a quiet atmosphere where people try to be as quiet as possible and reduce noise sources as much as possible, which helps infants to be more stable. This in turn results in less crying and fewer alarms. However, the theories presented here need to be confirmed by additional, more detailed studies.

In conclusion, this study demonstrated that integrating IMT and EMT modalities into an open bay NICU helped to reduce noise, which is crucial for infants’ well-being. Although both MT modalities resulted in significantly decreased noise levels, neither led to recommended noise levels of below 45 dBA. Both MT modalities resulted in a higher SNR compared to the control room, which implies that these modalities may be meaningful for the neurodevelopment of preterm infants. Additional studies that will evaluate sources of noise and sound in the NICU and investigate the influence of MT on specific noise generators are desirable to increase our understanding of the beneficial effects of MT in the NICU.

## Figures and Tables

**Table 1:** Patient characteristics.

Characteristic	Open Bay Room with MT	Open Bay Room without MT	*p* Value
Gestational age (weeks) *	31 ± 1.8	30 ± 2.8	0.28
Post-natal age at measurements (days) *	13 ± 6	11 ± 8	0.16
Ventilated infants (*n*) *	1.3 ± 0.9	1 ± 0.8	0.31
Infants per room (*n*) *	8 ± 2	7 ± 2	0.35
Caregivers present (*n*)	4 (1–7)	4 (2–7)	0.16
Duration of IMT sessions (min) *	49± 12	0	<0.001
Duration of EMT sessions (min) *	44 ± 9	0	<0.001

IMT—individual music therapy, EMT—environmental music therapy, * mean ± SD per session lasting approximately 45 min.

**Table 2:** Acoustic data.

Acoustic Parameter (Mean (SD) for all Recordings)	Open Room with MT	Open Room without MT	*p* Value	Effect Size *d* (95% CI)
Overall Leq (dBA)	53.1 (3.6)	61.4 (4.7)	0.02	2.1 (0.82, 3.42)
L90	49.2 (3.9)	55.4 (6.2)	0.03	1.8 (0.48, 3.19)
L50	54.2 (3.6)	57.6 (5.6)	0.06	0.5 (0.32, 0.74)
L10	58.9 (8.5)	66.4 (5.9)	0.03	1.7 (0.29, 2.6)
Average SNR *L*_50_ of events, dBA	18.1 (6.1)	10.3 (4.2)	0.01	2.8 (1.3, 3.86)
Occurrences of peaks ≥80 dBA/h	159 (29)	480 (52)	0.001	2.2 (0.8, 1.6)

Overall Leq—Average equivalent continuous noise level. L10, L50, and L90—A-weighted sound levels exceeding 10%, 50%, and 90% of the time, respectively. SNR—signal-to-noise ratio. CI—confidence interval, d—effect size (CI).

**Table 3:** Open room acoustic data for IMT and EMT.

Acoustic Parameter	IMT	EMT	*p* Value	Effect size (*d)* (95 CI)
Overall Leq dBA, mean (SD)	51.2 (4.2)	56.5 (4.1)	0.04	1.6 (0.53, 1.97)
L50 dBA, mean (SD)	50.1 (3.1)	55.6 (3.6)	0.04	0.7 (0.26, 1.42)
SNR L50 dBA, mean (SD)	19.2 (2.6)	17.6 (5.7)	0.05	0.3 (0.04, 0.93)

IMT—individual music therapy; EMT—Environmental music therapy; Overall Leq average equivalent continuous sound level; L10, L50, and L90—A-weighted sound levels exceeding 10%, 50%, and 90% of the time, respectively. SNR—Signal-to-noise ratio, CI—Confidence interval. Mean (SD).

## Data Availability

Data are available and will be given upon request.
